# Simulation-Based Curriculum in the Neonatal Intensive Care Unit (NICU): The New Era of Medical Education

**DOI:** 10.7759/cureus.86302

**Published:** 2025-06-18

**Authors:** Aashika Janwadkar, Akanksha Sancheti, Tracy Raschke, Dhaivat Shah, Vishakha C Nanda

**Affiliations:** 1 Neonatology, John H. Stroger, Jr. Hospital of Cook County, Chicago, USA; 2 Pediatrics, Rush Medical College, Chicago, USA; 3 Emergency Medicine, John H. Stroger, Jr. Hospital of Cook County, Chicago, USA; 4 Clinical Research/Family Medicine, Lower Bucks Hospital, Bristol, USA

**Keywords:** neonatal intensive care unit (nicu), neonatology fellows, simulation in medical education, simulations, training

## Abstract

Introduction

Simulations have been a cornerstone in teaching procedures and resuscitation algorithms in neonatal intensive care units (NICU). As advances in care and technology have reduced healthcare-related complications, simulations are being utilized to effectively increase confidence, link knowledge with clinical practice, and enhance procedural skills. The use of simulations in NICU training is on the rise, yet the quality and structure of neonatal simulations vary.

Objective

The objective is to enhance the self-efficacy and confidence of neonatal-perinatal medicine (NPM) fellows in managing common neonatal diseases in our NICU through simulation-based education.

Methods

A prospective educational cohort study was conducted in the NICU with NPM fellows. Fellows completed pre- and post-simulation questionnaires using a Likert scale. Each fellow completed six validated simulations based on NICU scenarios, with both pre-brief and debrief sessions. Statistical analyses were performed using SAS 9.4, with statistical significance defined as p<0.05. The difference between the pre- and post-intervention Likert scores was tested using the Wilcoxen rank-signed test.

Results

The study highlights positive shifts in five participants' confidence across diverse neonatal scenarios following their involvement in six simulations. Noticeable improvements are evident in symptom recognition (p<0.0001), scenario management (p<0.0001), NRP resuscitation skill (p=0.0029), code leadership (p=0.0004), and communication skills (p<0.0001). While p-values underscore statistical significance overall, adjusting for individual scenarios did not achieve statistical significance, due to the constraint of a smaller sample size.

Conclusion

Our study showed an overall positive impact on symptom recognition, scenario management, NRP resuscitation skills, code leadership, and communication skills, supporting the broader implementation of simulation-based training (SBT) in neonatology.

## Introduction

Simulation-based training (SBT) in medical education has evolved significantly, especially in high-acuity fields including neonatology. Training through simulation has become a fundamental aspect of education in many neonatal-perinatal medicine (NPM) fellowship programs. As highlighted in the 2021 European Resuscitation Council Guidelines, it serves as a cornerstone for teaching in neonatal resuscitation programs (NRP) and essential neonatal procedures, including “intravenous intraosseous and arterial cannulization, percutaneous long line insertion, lumbar puncture, and newborn hip examination” [1].

While there has been a rise in the quantity of simulation-based research (SBR) performed in the pediatric field, “the quality is highly variable” [2]. When developing SBR, it is important to consider several factors, including standardized validation, as these significantly impact the effectiveness of the educational intervention. However, there is still limited understanding of the steps needed to create the most effective SBR scenarios. 

Simulation-based technology has advanced to incorporate "high fidelity" training, which includes mannequins that offer trainees real-time feedback. Mannequins can be used to replicate complex clinical scenarios by exhibiting a range of symptoms, such as tachycardia and cyanosis, through features such as palpable pulses and color-changing skin [3]. Training now also includes confederates, or standardized patients, who assist by simulating family members or caregivers in neonatal scenarios, thereby creating a more realistic medical environment [2]. A study by Hossino et al. showed that high-fidelity simulation training in NRP improved self-confidence when performing neonatal intubation, running a resuscitation, treating neonatal respiratory arrest, performing chest compressions, and placing an umbilical venous catheter [4]. 

Although data on the direct impact of SBT on patient outcomes is limited, existing literature suggests that SBT has led to improvements in medical practice in multiple skills, enhancing clinical response to postpartum hemorrhage, reducing “time to needle” in stroke patients, and shortening time to blood transfusion in trauma patients [1]. 

Ghoneim et. al demonstrates that SBT for neonatology fellows may enhance both the short-term and long-term self-perceived effectiveness and knowledge related to the skills of communicating difficult news [5]. Research has also shown that repeated simulation-based practice not only improves performance and higher skill retention in comparison to conventional methods but also boosts collaboration, sharpens communication skills, and promotes more effective clinical decision-making [6]. SBT allows medical trainees to practice these skills in a controlled environment, facilitating constructive feedback and promoting reflective learning [7]. 

Effective simulation design and implementation can be achieved through standardized strategies, including scenario design, the use of confederates, and the incorporation of physical, conceptual, and emotional realism. Simulations can serve as an investigation methodology, with results applicable to clinical practice. In a study by Rostoker et al., simulations with a preterm mannequin were used to develop a validated scoring scale for providers practicing the least invasive surfactant administration (LISA) technique, based on video-recorded sessions [8]. Furthermore, structured debriefing and video capture can enhance the learning experience and facilitate the assessment of study outcomes [2].

The neonatal intensive care unit (NICU) is a challenging setting that demands proficiency in both technical and interpersonal skills to manage critically ill newborns effectively. The NICU team consists of a diverse multidisciplinary group, including nurses, nutritionists, child life specialists, and lactation consultants, each contributing their specialized expertise. Due to advancements in maternal-fetal medicine and improved neonatal care, complications seen in the NICU are decreasing but have not been eliminated. Thus, NPM fellows are required to be familiarized with these case scenarios or procedures. Given the necessity for medical team members to make rapid, life-saving decisions, SBT provides invaluable training for NPM fellows and their teammates, including the nurses, respiratory therapists, and advanced practice providers. SBT equips them to handle critical neonatal scenarios and to approach the emotional challenges of delivering difficult news in complex situations. 

We conducted this study to assess the impact of simulation-based education on NPM fellows’ self-efficacy and confidence in managing common neonatal diseases, and to design a validated simulation curriculum for our NICU.

## Materials and methods

This study is a prospective educational intervention cohort study done in a tertiary level (Level 3) NICU. The study involved multiple healthcare professionals, including nurses, fellows, physician assistants, nurse practitioners, and residents. 

Five NPM fellows participated in the study as research participants. Verbal consent was obtained from all participants prior to their inclusion in the study. Participants were informed about the research objectives, their roles, and the voluntary nature of their participation. Neonatal fellows were assigned a unique study code to ensure anonymity. Residents and advanced practice providers participated voluntarily, and their responses were not recorded for this study. The study was approved by the Institutional Review Board (IRB) of John H. Stroger, Jr. Hospital of Cook County (IRB Number: 23-025X).

Data for the study was collected using customized pre- and post-simulation questionnaires (Table 3 of Appendices). These questionnaires were developed specifically for the study and were modified to ensure clinical relevance to the educational content being delivered through the simulations. A seven-point Likert scale was designed with questions assessing participants’ perceptions of codes, symptom recognition, scenario management, NRP resuscitation skills, code leadership, and communication skills. They were validated by an attending physician and a mid-level provider not involved in the simulation study to ensure relevance and accuracy. The questionnaire assessed participants’ confidence in identifying and managing case content, self-efficacy, and self-perceived leadership qualities. 

Our study was designed using the deliberate practice framework by Ericsson to establish structured learning objectives and enhance goal-oriented training, while also incorporating the reflective practice framework by Schon to optimize simulations for individualized learning experiences and promote self-reflection during debriefing. These approaches are documented by the work of Zackoff et al. and the simulation guidebook by Soghier [9,10]. 

We designed simulations on real-life scenarios encountered in the NICU and they were validated by 10 independent neonatologists. The providers reviewed the clinical accuracy and relevance of the scenarios. Their feedback was discussed, disagreement was resolved with consensus and systematically incorporated into the final version of the study materials to ensure content validity. Reliability was supported through iterative revisions based on consensus among reviewers. A standardized checklist from the NRP 8th edition, which is publicly available and widely used for educational purposes in neonatal simulations, was used to ensure consistent settings and equipment across all simulations [11]. Each simulation included a pre-brief to introduce the case, followed by either high- or low-fidelity simulations. Simulations were performed in teams to mirror real-world collaboration, consisting of six mock codes, with each fellow leading a session or scenario. Each scenario was allotted 20-30 minutes including preparation time followed by a 15-20 minute debrief. We were guided by Kern’s model to create realistic cases for familiarizing the learners with the management of common NICU scenarios [9,10].

The checklist, which is publicly available and widely used for educational purposes in neonatal simulation, covers the following scenarios (Table 4 of Appendices): golden hour care for an extremely preterm infant (SIM 1), supraventricular tachycardia (SVT) and arrhythmias in a neonate (SIM 2), necrotizing enterocolitis (NEC) with shock (SIM 3), meconium aspiration in the delivery room with persistent pulmonary hypertension of the newborn (PPHN) (SIM 4), respiratory distress with pneumothorax or hemothorax (SIM 5), and delivering difficult news to families (SIM 6).

A structured debriefing session followed each simulation, focusing on learning objectives and allowing participants to reflect on their performance. Scripted educational material on each scenario was provided along with structured feedback to improve team performance. Specialty physicians were included during the feedback for additional inputs. 

Statistical analysis was performed on SAS 9.4. Independent variables included participants' years of experience and the type of simulation scenario, while dependent variables consisted of pre- and post-simulation confidence, self-efficacy, leadership qualities (measured using a Likert scale), and participant feedback. Statistical significance was defined as p<0.05. A descriptive analysis was performed of the feedback responses. The difference between the pre- and post-intervention Likert scores was tested using the Wilcoxen rank-signed test. 

## Results

Five participants (NPM fellows) took part in the pre- and post-questionnaire for the six simulations. Thus, a total of 30 responses were noted. All participants completed all the questions of the questionnaire. The mean of the years of neonatal experience including the time in fellowship for the participants recorded was four years. Two participants had five and 10 years of neonatal experience before the study. 

We assessed all 30 responses and compared them with the post-simulation questionnaire. Table 1 presents all the questions from the pre- and post-simulation assessments, along with the mean differences and corresponding p-values. Noticeable improvements are evident in symptom recognition (p<0.0001), scenario management (p<0.0001), NRP resuscitation skills (p=0.0029), code leadership (p=0.0004), and communication skills (p<0.0001) when aggregating all responses across all scenarios (Table 1).

**Table 1:** The mean pre- and post-participation questionnaire scores after participants engaged in SIMs. SIMs, six different simulations

Questions (Q)	Pre-participation score (N=30), mean (SD)	Post-participation score (N=30), mean (SD)	Mean difference	P-value
Codes scare me (Q3)	4.5 (1.25)	4.2 (1.69)	-0.3	0.3298
I feel confident in recognizing the signs and symptoms of the condition described in the simulation (Q4)	5.1 (1.14)	6.2 (0.71)	1.1	<0.0001
I am confident in my ability to manage the scenario described in the simulation (Q5)	5.1 (0.85)	6.2 (0.76)	1.1	<0.0001
I feel confident about performing neonatal resuscitation procedures (Q6)	5.8 (0.81)	6.2 (0.68)	0.4	0.0029
I feel confident to lead/supervise a code (Q7)	5.5 (0.63)	6.1 (0.68)	0.6	0.0004
I feel comfortable leading conversations with team members or family members (Q8)	5.4 (0.85)	6.1 (0.82)	0.8	<0.0001

We compared participants' perceptions of fear across all code scenarios. Overall, there was a decrease in fear levels post-simulation in SIM 1 (preterm infant delivery), SIM 2 (infant with SVT), SIM 3 (NEC with sepsis), and SIM 4 (infant with PPHN), with mean scores dropping from 4.83 to 4.17 in SIM 1, 4.6 to 4.2 in SIM 2, 4.8 to 4.0 in SIM 3, and 4.6 to 4.2 in SIM 4. However, in SIM 5 (chest tube insertion), the fear score increased from 4.0 to 5.0.

**Figure 1:**
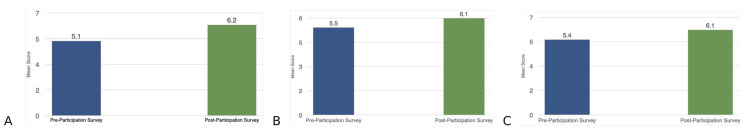
Representation of noticeable differences in various skills, including scenario management, code leadership, and communication skills. (A) Demonstrates noticeable differences in mean scores of pre- and post-simulation confidence in managing neonatal scenarios; (B) Represents improvement in mean scores of pre- and post-simulation confidence in code leadership; (C) Shows participants’ progress in mean scores of pre- and post-simulation comfort with communication skills.

Figure 1 describes the improvement seen in confidence in managing neonatal scenarios for individual SIMs. There is an increase of 1.1 points in the mean pre- and post-simulation scores. A similar improvement was observed, with a 0.6-point increase in the mean difference between pre- and post-simulation scores when participants were asked about their confidence in leading or supervising a code, and a mean increase of 0.8 points in their confidence when leading a conversation (Figure 1).

Table 2 illustrates the improvement in participants' self-efficacy and confidence scores across all scenarios following the simulations. The overall improvement in mean scores of participants before and after completing six simulations was 0.794. Overall, these changes reflect individual variations in learning and skill development following the simulations. 

**Table 2:** Improvement in participants' self-efficacy and confidence scores across all scenarios following the simulations.

	Pre-participation score (N=30), mean (SD)	Post-participation score (N=30), mean (SD)	Difference	p-value
Participant 1	5.9 (0.87)	6.7 (0.46)	0.8	<0.0001
Participant 2	5.6 (0.61)	6.8 (0.42)	1.2	<0.0001
Participant 3	5.0 (1.05)	5.6 (0.49)	0.6	0.011
Participant 4	5.5 (0.56)	5.6 (0.49)	0.1	0.343
Participant 5	4.9 (0.89)	6.2 (0.67)	1.3	<0.0001

Qualitative feedback by participants

Participants provided positive qualitative feedback on the simulations, stating, "A helpful experience that prepares you for similar situations. Overall, a great experience," and "Very realistic scenario, which was a helpful experience." They also appreciated the integration of equipment, noting, "Great simulation. I feel confident using the defibrillator machine. The debriefing was educational," and "The SVT code scenario increased my confidence." Regarding the quality of the debrief, participants shared, "The debrief was excellent and made me feel confident," and "The briefing effectively covered techniques, pros, and cons."

## Discussion

SBT has become essential in teaching NRP resuscitation and procedural skills in the NICU. As simulation technology continues to advance, its integration into residency and fellowship curricula is becoming increasingly common. SBT may help to enhance participant’s confidence and provides a safe environment for practicing patient management. As prenatal care improves and the incidence of complications decreases, incorporating SBT into fellowships offers a valuable way to build self-assurance and competence in managing uncommon but critical neonatal conditions. A study by Sawyer et al. demonstrated improvements in overall NRP performance scores and positive-pressure ventilation, through focused, repetitive, and well-defined simulation and feedback [12]. In our study, five NPM fellows participated. Other healthcare professionals, including nurses, respiratory therapists, APPs, and residents, were involved, although their responses were not collected. 

We gathered participants' perceptions of fear related to codes, symptom recognition, scenario management, NRP resuscitation skills, code leadership, and communication and procedural skills, with evidence of improvement across most areas in all scenarios, as shown in Table 1. A similar study by Simmons et al. found that NPM and cardiology fellows showed improved recognition of signs and symptoms of congenital heart disorders (CHD) after participating in simulations focused on infants with CHD [13].

We explored participants’ perceptions of their level of fear when involved in codes (Question 3 in Table 1). The mean score of pre-participation is 4.5, indicating a moderate level of fear. Post-participation, the mean score decreased to 4.2, suggesting a slight reduction in fear (p=0.32), which was not statistically significant. When individual simulations were analyzed, a decline in fear of codes was observed following participation in SIMs 1, 2, 3, and 4. These reflect a positive impact on participants' confidence and readiness. In contrast, fear levels increased post-participation in SIM 5 (chest tube insertion in infants with pneumothorax), indicating varied responses to different scenarios. This difference can be attributed to factors like individual previous experiences with codes and other situational variables. These findings underscore the complex relationship between simulations and participants’ fear levels, emphasizing the importance of individually tailored training approaches. A study examining self-efficacy and performance in chest compressions and bag-valve-mask ventilation found a positive correlation between previous experience with codes and mock codes and self-efficacy [14].

We carefully designed our simulations and learning objectives in alignment with Sweller’s cognitive load theory to enhance learning effectiveness and prevent any negative impact on learners [9,10]. The simulations were chosen from various subspecialties in the NICU to provide a variety of cases to the participants. With the participant's consent, the simulation was video recorded for performance grading and providing constructive feedback. This approach, employed in previous SBR including a study by Sawyer et al., serves as an effective grading tool [12]. The debriefing was structured to allow fellows to express their thoughts, followed by the delivery of constructive feedback [2,10]. An educational PowerPoint was reviewed after each simulation regarding the case’s pathophysiology. We modified our debriefing using the experiential learning theory proposed by Gibb’s reflective cycle [10]. 

Efforts were made to involve subspeciality faculty during the debriefing. During the simulation of a neonate with SVT, a trained ER physician demonstrated the proper setup and use of a defibrillator on a neonate. In the SIM designed to demonstrate the delivery of difficult news, a palliative care faculty member participated in both the simulation session and debriefing. The inclusion of NICU and palliative care attendings was particularly valued for their feedback. 

Some participants shared qualitative feedback for more simulations with faculty involvement: “I loved the fact that NICU and palliative care attending were present during the feedback, this was helpful.” Another participant noted, “We need more simulations of difficult news. Also, if faculty can demonstrate how they will deliver difficult news in such scenarios can be helpful.” 

Auerbach et al. state that a repetitive pattern of simulation (simulation-debrief-simulation) was more helpful than a standard technique of simulation followed by a debriefing [15]. A study by Ghoneim evaluated delivering bad news using SPIKES protocol via a simulation and followed the intervention again in three to four months, and they concluded that repetitive administration of simulation can increase short- and long-term retention methods, thus there is a need for longitudinal curriculum in training [5]. Another study by Janice et al. demonstrated that incorporating a simulation-based curriculum for delivering difficult news into the neonatal training program enhanced the comfort level of neonatal fellows in handling such conversations [16]. These findings align with feedback from our participants, who expressed strong support for including simulations in the curriculum. They commented, "These simulations helped me be more confident in my management of these critical cases. I vote to have it incorporated into NICU interval mock code practice." Another fellow shared, "SIM sessions were useful and practical. Some cases are rarely seen on a daily basis, so SIM gives the experience needed to manage similar cases effectively." 

We had two fellows with 10 and five years of experience, respectively. We observed improvements in self-confidence and self-efficacy across all scenarios, with all participants showing positive progress. Notably, Participants 1, 2, 3, and 5 demonstrated significant improvements, as indicated by significant p-values (Table 2). These findings align with Donhue's study, which also highlighted a connection between self-efficacy and experience [14].

In the post-evaluation questionnaire, we asked for participants’ feedback. The feedback highlights that the simulations were well-organized, realistic, and instrumental in building participants' confidence and skills. Participants appreciated having actual equipment available, minimizing assumptions, and found the debriefing sessions to be educational and systematic. Many noted increased confidence, especially with handling defibrillators and complex scenarios. One comment for the SVT scenario involving the use of a defibrillator was “Good scenario as we do not see such cases often, helps revise the basics and keeps knowledge updated. Very systematic debrief.” 

Our study had some limitations. The small sample size was due to the limited number of fellows, though efforts are underway to involve residents in future phases. Additionally, there was a lack of high-fidelity mannequins, particularly for preterm infants, which could have enhanced the simulation experience. Furthermore, we were unable to repeat the simulations to assess whether retention over time would impact participants' confidence and self-efficacy. 

Despite these challenges, the study had several strengths. It featured neonatal scenarios involving multiple specialties, making it, to our knowledge, the first paper to focus on multiple specialty simulations modeling a simulation curriculum for fellowship. All the scenarios were validated by attending physicians. Each session included detailed debriefs with educational material and active faculty involvement. The feedback from participants emphasized the value of SBT in building confidence and preparing them for real-life scenarios. Current literature provides guidance to educators for developing standardized SIM models and debriefing. We utilized these guidelines to achieve learner objective goals and tailored learning experiences [9,10].

Although validating these results and ensuring generalizability is challenging due to the study's small sample size and single institution setting, its potential impact remains significant. We acknowledge the limitations of the study and thus conducting this study on a larger scale would allow learners to get exposed to a wider spectrum of standardized scenarios, offering fellows broader clinical exposure and enhancing their training experience. To summarize, though the scope of simulation in neonatal medicine is growing, the quality of simulation varies and there is insufficient data in the literature on effective structural designs for neonatal simulations.

## Conclusions

The study demonstrated an overall positive impact on various management and resuscitation skills among NPM fellows, despite the limitations of the small sample size. It supports the need for simulation-based curriculum development as a requirement in neonatology. Expanding the simulation-based curriculum and enhancing SBR to explore a broader range of scenarios and situational factors influencing participant outcomes is essential, especially as advancements in care and technology have reduced prematurity-related and healthcare-related complications while improving the prognosis of preterm neonates during the perinatal and postnatal periods. Simulations play a critical role in this progress by boosting confidence, bridging knowledge with clinical practice, and enhancing procedural skills effectively.

## Appendices

**Table 3:** The pre- and post-participation surveys used in the study.

Pre-Participation Survey: Following is the questionnaire regarding our study; we hope to have your sincere opinion while answering the same. Participation in simulations as well as surveys is voluntary. Participants can withdraw from the study at any time. Participation or non-participation in this research will have no effect on their employment.								
Q1	Participant’s study code:							
Q2	Years of experience in neonatology:							
Q3	Codes scare me. Using a scale of 1-7 (1=Strongly Disagree and 7=Strongly agree), How would you rate the following statements?	1 Strongly Disagree	2 Mostly disagree	3 Somewhat disagree	4 Neither agree nor disagree	5 Somewhat agree	6 Mostly agree	7 Strongly Agree
Q4	I feel confident to recognize the signs and symptoms of the condition described in pre-brief of the simulation. Using a scale of 1-7 (1=Strongly Disagree and 7=Strongly agree), How would you rate the following statements?	1 Strongly Disagree	2 Mostly disagree	3 Somewhat disagree	4 Neither agree nor disagree	5 Somewhat agree	6 Mostly agree	7 Strongly Agree
Q5	I am confident in my ability to manage the scenario described in pre-brief of the simulation. Using a scale of 1-7 (1=Strongly Disagree and 7=Strongly agree), How would you rate the following statements?	1 Strongly Disagree	2 Mostly disagree	3 Somewhat disagree	4 Neither agree nor disagree	5 Somewhat agree	6 Mostly agree	7 Strongly Agree
Q6	I feel confident about performing neonatal resuscitation procedures (possibly required in this scenario). Using a scale of 1-7 (1=Strongly Disagree and 7=Strongly agree), How would you rate the following statements?	1 Strongly Disagree	2 Mostly disagree	3 Somewhat disagree	4 Neither agree nor disagree	5 Somewhat agree	6 Mostly agree	7 Strongly Agree
Q7	I feel confident to lead/supervise a neonatal code. Using a scale of 1-7 (1=Strongly Disagree and 7=Strongly agree), How would you rate the following statements?	1 Strongly Disagree	2 Mostly disagree	3 Somewhat disagree	4 Neither agree nor disagree	5 Somewhat agree	6 Mostly agree	7 Strongly Agree
Q8	I feel comfortable in leading conversations with team members or family members. Using a scale of 1-7 (1=Strongly Disagree and 7=Strongly agree), How would you rate the following statements?	1 Strongly Disagree	2 Mostly disagree	3 Somewhat disagree	4 Neither agree nor disagree	5 Somewhat agree	6 Mostly agree	7 Strongly Agree
Post-Participation Survey: Following is the questionnaire regarding our study; we hope to have your sincere opinion while answering the same. Participation in simulations as well as surveys is voluntary. Participants can withdraw from the study at any time. Participation or non-participation in this research will have no effect on their employment.								
Q1	Participant’s study code:							
Q2	Years of experience in neonatology:							
Q3	Codes scare me. Using a scale of 1-7 (1=Strongly Disagree and 7=Strongly agree), How would you rate the following statements?	1 Strongly Disagree	2 Mostly disagree	3 Somewhat disagree	4 Neither agree nor disagree	5 Somewhat agree	6 Mostly agree	7 Strongly Agree
Q4	I feel confident to recognize the signs and symptoms of the condition described in pre-brief of the simulation. Using a scale of 1-7 (1=Strongly Disagree and 7=Strongly agree), How would you rate the following statements?	1 Strongly Disagree	2 Mostly disagree	3 Somewhat disagree	4 Neither agree nor disagree	5 Somewhat agree	6 Mostly agree	7 Strongly Agree
Q5	I am confident in my ability to manage the scenario described in pre-brief of the simulation. Using a scale of 1-7 (1=Strongly Disagree and 7=Strongly agree), How would you rate the following statements?	1 Strongly Disagree	2 Mostly disagree	3 Somewhat disagree	4 Neither agree nor disagree	5 Somewhat agree	6 Mostly agree	7 Strongly Agree
Q6	I feel confident about performing neonatal resuscitation procedures (possibly required in this scenario). Using a scale of 1-7 (1=Strongly Disagree and 7=Strongly agree), How would you rate the following statements?	1 Strongly Disagree	2 Mostly disagree	3 Somewhat disagree	4 Neither agree nor disagree	5 Somewhat agree	6 Mostly agree	7 Strongly Agree
Q7	I feel confident to lead/supervise a neonatal code. Using a scale of 1-7 (1=Strongly Disagree and 7=Strongly agree), How would you rate the following statements?	1 Strongly Disagree	2 Mostly disagree	3 Somewhat disagree	4 Neither agree nor disagree	5 Somewhat agree	6 Mostly agree	7 Strongly Agree
Q8	I feel comfortable in leading conversations with team members or family members. Using a scale of 1-7 (1=Strongly Disagree and 7=Strongly agree), How would you rate the following statements?	1 Strongly Disagree	2 Mostly disagree	3 Somewhat disagree	4 Neither agree nor disagree	5 Somewhat agree	6 Mostly agree	7 Strongly Agree
Q9	Feedback for Scenario, experience during simulation and debrief:							

**Table 4:** Brief overview of the six simulation scenarios.

Simulation 1: Golden hour care for extremely preterm infant	
Brief summary of the case	Birth of a very low birth weight (VLBW) baby
Learner’s action checklist	1. Preparing for the preterm labor
	2. Neonatal resuscitation program (NRP) training in the delivery room
	3. Managing the components of golden hour
	4. Admitting the neonate to NICU and initial orders
Brief H&P	Baby boy to be born via C-section at 26 weeks to 24-year-old G3P1011 who is presenting with preterm labor and bulging bags; prenatal: s/p betamethasone (BMZ) yesterday and today, magnesium sulfate drip for neuro prophylaxis given for last 24 h; group B streptococcus (GBS) status unknown, received adequate penicillin (PCN) prophylaxis; prenatal labs: within normal limits (WNL); NICU consult was done yesterday, mother desires full resuscitation.
Initial vital signs (after stabilization):	Temperature: 37.6, heart rate: 160, respiratory rate: 50, blood pressure: 40/20, oxygen saturation: 93%, birth weight (BW): 750 g
Initially physical exam (after stabilization)	General: Alert and moving. HEENT: Eye open sometimes, anterior fontanelle is soft and open. Neck: Normal. Lungs: Bilateral equal air entry; bilateral intercostal retractions+; air entry equal bilaterally. Cardiovascular: No murmurs. Good pulses. Abdomen: Normal. Neurological: Extremities full range of motion. Skin: Baby is noted to have thin skin, some generalized bruises noted.
Current event in SIM: (these events are a model for SIM coordinator and are subject to change as per participant’s decision during the SIM)	
In the delivery room - the infant was limp, with no respiratory effort; brought to warmer bed, covered with plastic wrap over the heating mattress, head cap placed, suctioned, CPAP started; intubated at 5 minutes of life. FiO2 adjusted for target saturations (initial FiO2 30%). Neo puff over endotracheal tube ventilated. Surfactant given. Apgar 3 (color 0, pulse 2, grimace 1, tone 0, respiration 0) / (color 1, pulse 2, grimace 2, tone 1, respiration 1) 7 @ 1- and 5-min, birth weight (BW) 750 grams. Baby transported to NICU on transport bed on synchronized intermittent mandatory ventilation (SIMV) RR 40, PIP 17, PEEP 5, FiO2 50% (setting vary as per participants). On admission - temperature on admission 37.6°C. Peripheral IV unable to obtain. Umbilical catheter placement done. IVF and antibiotics started within an hour. Initial orders placed.	
Simulation 2: Supraventricular tachycardia (SVT) and arrhythmias in a neonate	
Brief summary	A term infant is having tachycardia (190-240). Nurse calls you at bedside.
Learner’s action checklist	1. Obtain and interpret a 12-lead EKG in a neonate with tachycardia
	2. Differentiate wide from narrow complex tachycardia
	3. Use vagal maneuvers and administer adenosine; can be repeated
	4. Demonstrate correct technique for synchronized cardioversion
	5. Recognize the role of sedation for cardioversion
Brief H&P	Female newborn born to a 39-year-old G9P1435 at 39 weeks GA, born via stat C-section for non-reassuring fetal heart rate (NRFHT). BW - 3600 g. Most prenatal labs are pending, except HIV negative and EIA negative. Prenatal ultrasound all WNL. A fetal echo was done due to advanced maternal age (AMA). Normal fetal echo. APGARS 5 and 7 at 1 and 5 minutes of life. Baby admitted to NICU in lieu of risk of sepsis. On ampicillin and gentamicin. At 12 hours of life, neonates developed persistent tachycardia. The nurse calls you bedside to evaluate the infant.
On exam	Temperature 37, heart rate 300 (rhythm is with a narrow complex - SVT), respiratory rate 50, blood pressure 60/40, oxygen saturation 98%; on room air. Calm and sleeping. Peripheral IV (PIV) in right forearm.
Initial physical exam	General: No distress, sleeping. HEENT: Normal cephalic, atraumatic, anterior fontanelle is soft and open. Neck: Normal. Lungs: Clear lungs. Cardiovascular: Tachycardic. No murmurs. Good pulses. Abdomen: Normal. Neurological: Normal. Skin: Normal. GU: Normal.
Events during the SIM: (These events are a model for SIM coordinator and are subject to change as per participant’s decision during the SIM)	
At 12 hours of life, neonate developed persistent tachycardia with heart rate above 200 (210 to 220). The infant is calm and sleeping on exam. Participants ask for EKG strip. On the monitor, the strip looks like a narrow complex tachycardia. Nurse gets the EKG. Meanwhile, participants may try vagal maneuver (ice on face) or wait for EKG. Other vitals at this point are stable. (Vitals: Temperature 37, Heart Rate 300 (rhythm is with a narrow complex - SVT), Respiratory Rate 50, Blood pressure 60/40, Oxygen saturation 98%). Adenosine 0.1 mg/kg IV given along with vagal maneuvers (ice on face). (Vagal maneuver can be done multiple times. Adenosine can be repeated). Neonate had poor response and continues to be tachycardic with heart rates in the >250s blood pressure. 5 mins later infant continues tachycardic, looks pale, infant irritable, and crying. Participants should ask for another set of vitals. Blood pressure noted to be 50/30, infant has weak pulses on exam. (Vitals: Temperature 37, Heart Rate 320 (rhythm is with a narrow complex), Respiratory Rate 70, Blood pressure 50/30, Oxygen saturation 89%). Participants ask for code crash cart to do a cardioversion. Sedation with Fentanyl to be done prior to cardioversion. (Nurse can prompt if participant does not ask for it). Pads placed and joules adjusted to be given. Synchronized cardioversion with 1 J/kg performed, which successfully returned to sinus rhythm with heart rate in the 160s to 170s beats per minute. Alternate - Participants can also stabilize the airway prior to cardioversion with endotracheal tube.	
Simulation 3: Necrotizing enterocolitis (NEC) with shock	
Brief summary of the case	A 30-day-old preterm infant is having hypotension and bradycardia, along with Increased respiratory requirement. Nurse calls you at bedside.
Learner’s action checklist	1. Identification of septic infant
	2. Stabilizing the patient (ABCD)
	3. Identifying necrotizing enterocolitis (NEC) as a cause of sepsis
	4. Initial management of NEC
Brief H&P	A 30-day-old female infant born to a 28-year-old G6P1132 at 25+3 weeks GA preterm, appropriate for gestational age. Complications of pregnancy include no prenatal care and anemia. No history of medications during pregnancy. Maternal social history is significant for cigarette and marijuana smoking. Missing prenatal labs initially. Available prenatal labs include: COVID-19 negative, HIV negative, and Hepatitis B negative. Mom presented to OSH in premature labor with a bulging sac and transverse fetal lie. Medications during L&D included magnesium sulfate drip, betamethasone x1. Neonate was born via emergency C-section. APGARS were 4 and 8 at 1 and 5 minutes. The infant was intubated at 10 min of life. Surfactant given. Neonate was transferred from outside hospital.
Vital signs	Temperature 37, heart rate 90, respiratory rate 80, blood pressure 40/30, oxygen saturation 89-92%; BW was 570g; today’s weight=1100 grams.
Respiratory setting	On non-invasive positive pressure ventilation (NIPPV): RR 25, PIP 18, PEEP 5, PS 6, FiO2 35%.
Appearance	Tired looking
Lines	Peripherally inserted central catheter (PICC)
Initial Physical exam	General: Pale and lethargic, nasal cannula in place for NIPPV. HEENT: Normal cephalic, atraumatic, anterior fontanelle is soft and open. Neck: Normal. Lungs: Tachypnea, suprasternal retractions, clear lungs. Cardiovascular: No murmurs or weak pulses. Abdomen: Distended, guarding, dusky discoloration of abdomen, breath sounds absent. Neurological: Normal, sleepy, poor suck. Skin: See abdomen. GU: Normal.
Current event in SIM: (These events are a model for SIM coordinator and are subject to change as per participant’s decision during the SIM)	
On DOL 30; infant while on NIPPV is noted to have increased work of breathing. Participants ask for vitals: hypotension to 40/30, temperature 37, heart rate 160, respiratory rate 80, blood pressure 40/30, oxygen saturation 89-92%, current weight 1100 g. Feeds are held and nasogastric tube to low intermittent suction. Infant was on full feeds. IV fluids started. Blood culture, CBC, and CRP sent. Gas obtained. Participants can try 1 bolus NS at 20 ml/kg. Infant will be still hypotensive. Neonate started on dopamine for hypotension. Gas and CBC values (WBC 28.3, Hb 11.4, Hct 33.9, Plat 469, N=72.3, Bands=5; pH shows 7.277, PCO2 48.5, pO2 58, HCO3 18.4, BE -3.0). Due to continuation of worsening shortness of breath, desaturations (70-80), lethargic appearance, and now new onset of bradycardia (80-90), infant intubated and started on synchronized intermittent mandatory ventilation (SIMV) RR 40, 20/6, PS 6, IT 0.4, FiO2 40%. Antibiotics to be started. Abdominal X-ray flat plate ordered. X-ray showed that baby has NEC with air under diaphragm. Call surgeons. Update parents.	
Simulation 4: Meconium aspiration in the delivery room with persistent pulmonary hypertension of the newborn (PPHN)	
Brief Summary of the case	Called for a level 3 delivery due to PROM (prolonged rupture of membrane) and Meconium-stained amniotic fluid and non-reassuring fetal heart rate (NRFHT).
Learner’s action checklist	Identifying surfactant need in Meconium aspiration syndrome
	Switching to high-frequency oscillatory ventilation (HFOV) to optimize ventilation in PPHN
	Maintaining blood pressure
	Getting an ECHO and identifying PPHN treatment
	When to transfer for ECMO (extracorporeal membrane oxygenation)
Brief H&P, pertinent maternal and neonatal labs, and past medical history	Infant born at 41.1 weeks via C-section. BW 3050 g. Complications during delivery included PROM and NRFHT. Rupture of membranes ~25 hours, amniotic fluid meconium stained. APGARs were 1, 2, and 4. Initial endotracheal placement was successful. Prenatal labs: O+, Ab-, rubella immune, HBsAg NR, HCV Ab NR, RPR NR, HIV NR, GBS positive, received penicillin x2. Can ask for cord arterial gas: pH 7.2 / pCO2 60 / PaO2 37 / HCO3 16 / BE -8. On cooling blanket. On sedation.
Initial Vital signs: lines	Temperature 37, heart rate 160, respiratory rate 80, blood pressure 60/40, O2 saturation 89%. UV and left radial line.
Current respiratory status/setting	Intubated
Overall appearance	Respiratory distress
Initial physical exam	General: moderate respiratory distress. HEENT: normal cephalic, atraumatic, anterior fontanelle is soft and open. Neck: normal. Lungs: tachypneic, suprasternal retractions with nasal flaring, clear lungs. Cardiovascular: pale, no murmurs, weak pulses. Abdomen: normal. Neurological: normal. Skin: normal. GU: normal.
Current event in SIM: (These events are a model for SIM coordinator and are subject to change as per participant’s decision during the SIM)	
When admitted to the NICU, the patient was on SIMV with saturations 70-75% with 75-80% FiO2. Synchronized Intermittent Mandatory Ventilation (SIMV) RR 45, PIP 25, PEEP 5, PS 6, iT 0.45, FiO2 75%. Vitals: Temperature 37, Heart Rate 160, Respiratory Rate 80, Blood Pressure 60/40, Oxygen saturation 89%. Participants can ask for gas, CBC, blood culture, CXR, and start baby on Ampicillin and Gentamicin. Can get central line or peripheral arterial line. Initial arterial gas: pH 7.1 / PCO2 68.4 / PO2 63 / HCO3 9.3 / BE -16, Na: 135, K: 4.0, iCa: 1.56, Glu: 192, Hct: 46%. OI is 15 (mean airway pressure with MAP 12). WBC 18.3, Hb 11.4, Hct 33.9, Plat 469, N=42.3, Bands=5. X-ray showed hazy bilateral opacities/lung fields. Endotracheal tube is in good place. Chest expansion 8 ribs. The baby may receive a dose of surfactant. Participants might ask for an increase in FiO2 or rate. Due to distress and currently on 100% SIMV and desaturating to 80s%; transitioned to HFOV with initial MAP of 14, frequency 10, amplitude 33. After starting the HFOV, the saturations improved to 90s briefly. (This is a model, setting and OI can change as per participant’s decision.) Infant continues to desaturate after 15-30 mins to 75-80% on 100% FiO2. Participants can ask for a gas and ECHO. (Can ask for ECHO.) Arterial gas: pH 7.13 / PCO2 46 / PO2 56 / HCO3 10.4 / BE -15. OI of 25 (with MAP 14). Get a CXR. Can change vent settings. ECHO done showed elevated right-sided cardiac pressure, suggesting PPHN and normal LV function. Inhaled Nitric Oxide (iNO) started at 20 ppm. The patient was also having low blood pressure, started on Dopamine at 10 mcg/kg/hr. Received NS bolus. Vitals: Temperature 37, Heart Rate 160, Respiratory Rate 65, Blood Pressure 40/20, Oxygen saturation 82%. Improved the saturations and were sustained in 90-95% FiO2, improved blood pressure. After 30 mins, the baby started desaturating with low blood pressure, while on dopamine. Participants ask for a gas, CXR. Latest gas: pH 7.2 / PCO2 37 / PO2 50 / HCO3 14 / BE -12. MAP of 16, frequency 10, amplitude 33, FiO2 95%. OI is 30. Dobutamine was added and ECMO center was contacted. Parents updated.	
Simulation 5: Respiratory distress with pneumothorax or hemothorax	
Brief summary	A term infant is having respiratory distress. Your nurse calls you.
Learner’s action checklist	1. Identify respiratory distress
	2. Identify pneumothorax as a possible cause, on physical examination and chest X-ray finding
	3. Management of pneumothorax and reassess
	4. When to place chest tube and its procedure
	5. Sedation prior to chest tube
Brief H&P, pertinent maternal and neonatal labs, and past medical history	Baby boy born via routine repeat C-section at 38.5 weeks. Nuchal cord x 1. Baby cried weakly soon after birth and was brought to the radiant warmer. The baby was then noted to have apnea. Routine care was given, with suctioning from mouth and nose. Thick meconium suctioned from the mouth. HR was < 100, so Positive Pressure Ventilation (PPV) started. Continued for about 3 mins, then HR > 100, and baby started to cry spontaneously. Respiratory distress noted with grunting and subcostal retractions. Continued CPAP +6 at FiO2 60%. APGARs were 5 (Color -1, HR -1, Resp -1, Tone -1, and Grimace -1) and 9 (1 off for respiration) at 1 and 5 mins of life, respectively. Baby's transfer to NICU on CPAP for further management. Started with Ampicillin and Gentamicin. CBC and blood culture sent. Chest X-ray ordered.
Initial vital signs	Temperature 37, heart rate 160, respiratory rate 80, blood pressure 70/40, oxygen saturation 82-98%. BW 3060 g.
Current respiratory status/setting	On CPAP + 6, FiO_2_ 40%
Overall appearance	Agitated
Lines	L hand PIV
Initially physical exam	General: in moderate respiratory distress, grunting, and nasal flaring. HEENT: normal cephalic, atraumatic, anterior fontanelle is soft and open. Neck: normal. Lungs: tachypneic and suprasternal retractions, decreased air entry on right side. Cardiovascular: no murmurs. Abdomen: normal. Neurological: WNL. Skin: normal. GU: normal. Psychiatric: normal.
Current event in SIM: (These events are a model for SIM coordinator and are subject to change as per participant’s decision during the SIM)	
Infant evaluated at bedside at 2 hrs of life, with RR 80, HR 160 with increasing O2 requirement to now 100% FiO2 on CPAP of +6. SpO2 92%, blood pressure 60/40. On exam, baby is grunting and retracting. Participants ask for chest X-ray (CXR). Baby is already on Ampicillin and Gentamicin. CBC results are as below from admission (WBC 18.3 Hb 11.4 Hct 33.9 Plat 469, N=42.3, Bands=0). Blood culture in process. Participants can add C-reactive protein and blood gas. CXR shows right pneumothorax. pH 7.277 PCO2 54.5 pO2 58 HCO2 25.4 BE -1.0. Vitals deteriorate (decrease blood pressure and HR). Needle decompression done, mild improvement but continues to be in distress. Repeat vitals showed blood pressure 50/30 with HR 90 and SpO2 89%. Baby intubated and settings changed to RR 35 PIP 20, PEEP 4, FiO2 80-100%. (Intubation is optional, depends on the participant and progression of the case). Can order repeat CXR or judge clinically. Fentanyl/other sedative agent given and then chest tube placed. Infant’s vitals and blood gas improve. Repeat CXR improved.	
Simulation 6: Delivering difficult news to families	
Brief summary	Delivering difficult news to a family of a neonate with multiple anomalies.
Scenario/brief H&P	For the fellow: Baby boy Sanchez is a 34-week infant with prenatally undiagnosed trisomy 18 (T18) with dysmorphic features, birth weight 1200 kg, who has microcephaly, micrognathia, rocker-bottom feet. The infant was born to a 46-year-old G1P0 mother, with advanced maternal age (AMA). She had good prenatal care and was expecting a healthy baby. After delivery, he was noted to have dysmorphic features and worsening respiratory distress. The infant is admitted to NICU, currently intubated. It is now day of life (DOL) 6 and multiple tests have come back. The infant failed an extubation attempt on DOL 1 due to apnea and worsening acidosis, and is now on the ventilator with extremely high pressures and minimal spontaneous respiration. The infant has severe pulmonary atresia (prostaglandin dependent) and evidence of persistent pulmonary hypertension of newborn (PPHN) on echo. The FISH returned positive for T18 this morning and cardiac surgery states that surgical intervention would be futile given the T18 diagnosis and the PPHN on echo making cardiac surgery impossible. You have been told the mother is very invested, sits at the bedside 24 hours a day, jumps on every alarm at the bedside, and very much wants this child. Dad does not visit much. Given the diagnosis and poor prognosis, the care team feels care should be redirected. Your job is to plan this meeting and have this difficult conversation.
For the simulated patient (SP) Mom	You are 46 years old, and your baby, John, has been diagnosed with several medical problems. He needs a ventilator to help him breathe and has a heart defect that requires surgical intervention. John also has a small head, a small jaw, and unusual hands and feet. You are aware that a genetic test for trisomy 18 was ordered and should provide results today. Also, the heart surgery team is scheduled to discuss repairing his heart. The medical team has explained that if John has trisomy 18, his life may be significantly shortened, and he is likely to have profound developmental disabilities. Despite this, your love for him. You want him to have every chance, and you spend hours by his bedside, watching him.
Questions/expressions for SP mom (more hopeful and sad tone)	With advancement in medicine, can't we do anything?
	I feel the surgery will be a miracle
	I have been praying every day, and I know miracles can happen. I want you to try everything.
	I know the saturations drop because of his heart issue and it will resolve with surgery
	I will pray hard for his post-surgery course to be easy.
	John’s grandparents are excited to see him at home.
	I did everything right during the pregnancy and saw all the doctors and the baby was supposed to be healthy.
For the simulated patient (SP) Dad:	You are 50 years old. As the devastating news about John’s severe medical issues and likely short, painful life emerged, you found yourself withdrawing. Your wife is so sad, and you do not know how to help. You do not want to visit because he looks like he is suffering with all the tubes and wires. You are angry and you find yourself wishing this painful situation could just be over.
Questions for SP Dad: (more of angry and refusing kind tone)	What do you mean that surgery cannot happen? This is a big hospital with a good name and so we came here.
	John is suffering with the tube and all the medications he is getting; can you not fix what is happening to him?
	We want our baby to be transferred to another hospital?
	He is our miracle child, what do you mean by “let him go” or term used?
Initial vital signs:	Temperature 37, Heart Rate 170, Respiratory Rate 50, Blood pressure 60/40, Oxygen saturation 98%
Current respiratory status/setting	Intubated
Overall appearance	Calm and sleeping
Lines	Peripheral IV Left arm; Umbilical vein
Physical exam	As mentioned in the vignette
